# 151 The Impact of a community based exercise rehabilitation program (ExWell Medical) on people with diverse chronic illnesses

**DOI:** 10.1093/eurpub/ckae114.114

**Published:** 2024-09-26

**Authors:** Fiona Skelly, Noel McCaffrey, Emmett Byrne, Andrew McCarren, Seán P Kennelly

**Affiliations:** ExWell Medical, IWA Clontarf, Ireland; SHE Research Group, Department of Sport and Health Sciences, Technological University of the Shannon, Athlone, Ireland; ExWell Medical, IWA Clontarf, Ireland; ExWell Medical, IWA Clontarf, Ireland; School of Computing, Dublin City University, Ireland; Department of age-related healthcare, Tallaght University Hospital, Ireland; Department of Medical Gerontology, School of Medicine, Trinity College Dublin, Ireland

## Abstract

**Background:**

Chronic diseases (CD) represent a substantial and growing burden on health care services globally and are the current leading cause of morbidity and mortality. Exercise rehabilitation has the potential to enhance functional capacity and quality of life (QoL) for those living with CD, regardless of specific condition. Community-based exercise rehabilitation (CBER) programs offer a highly scalable and feasible means of extending the outreach of exercise rehabilitation for the CD population. The aim of this study was to investigate the effects of 12 weeks of community-based exercise rehabilitation program (CBER) on individuals with a range of CDs.

**Methods:**

Adults with a range of CD were enrolled in a CBER (ExWell Medical). Measurements of body composition, physical functioning and health-related quality of life were conducted at three timepoints; baseline, 6 wks and 12 wks. Participants attended 2 classes per week for the duration of the study. Each class was 45 mins in duration and included a combination of aerobic, resistance and balance training.

**Results:**

A total of 310 participants (mean age 70.0 ± 11.2 yrs; 45.1% male) were recruited for this study. Significant improvements were observed in body composition (p = 0.001, Cohen’s D = 0.02), cardiorespiratory fitness (p < 0.001, Cohen’s D = 0.74), lower body strength (p < 0.001, Cohen’s D = 0.32), functional mobility (p < 0.001, Cohen’s D = 0.08), and self-rated health (p < 0.001, Cohen’s D = 0.06). The majority of improvements were achieved by the 6 wk assessment and maintained at 12 wks. Individuals with lower levels of cardiorespiratory fitness at baseline exhibited a greater percentage improvement at 12 weeks.

**Conclusion:**

The ExWell Medical CBER is an effective approach to the secondary prevention of CD. Clinically meaningful improvements in physical functioning can be achieved within as little as 6 wks. Adopting an integrated approach to exercise rehabilitation, has the potential to widen the access of this service to the growing CD population in Ireland.

